# Geometry Reduced Order Modeling (GROM) with application to modeling of glymphatic function

**DOI:** 10.1016/j.brainresbull.2025.111558

**Published:** 2025-10-15

**Authors:** Andreas Solheim, Geir Ringstad, Per Kristian Eide, Kent-Andre Mardal

**Affiliations:** a Department of Numerical Analysis and Scientific Computing (SCAN), Simula Research Laboratories, Kristian Augusts gate 23, Oslo, 0164, Norway; b Department of Mathematics, University of Oslo, Moltke Moes vei 35, Oslo, 0851, Norway; c K.G. Jebsen Centre for Brain Fluid Research, University of Oslo, Pb 1072 Blindern, Oslo, 0316, Norway; d Department of Radiology, Oslo University Hospital, Pb 4950 Nydalen, Oslo, 0424, Norway; e Institute of Clinical Medicine, University of Oslo, Klaus Torgårds vei 3, Oslo, 0372, Norway; f Department of Geriatric Medicine, Sørlandet Hospital Trust, Pb 416 Lundsiden, Arendal, 4604, Norway; g Department of Neurosurgery, Oslo University Hospital-Rikshospitalet, Sognsvannsveien 20, Oslo, 0372, Norway

**Keywords:** Model order reduction, Glymphatic system, Idiopathic Normal Pressure Hydrocephalus, Numerical simulation, Image registration

## Abstract

Computational modeling of the brain has become a key part of understanding how the brain clears metabolic waste, but patient-specific modeling on a significant scale is still out of reach with current methods. We introduce a novel approach for leveraging model order reduction techniques in computational models of brain geometries to alleviate computational costs involved in numerical simulations. Using image registration methods based on magnetic resonance imaging, we compute inter-brain mappings which allow previously computed solutions on other geometries to be mapped on to a new geometry. We investigate this approach on two example problems typical of modeling of glymphatic function, applied to a dataset of 101 MRI of human patients. We discuss the applicability of the method when applied to a patient with no known neurological disease, as well as a patient diagnosed with idiopathic Normal Pressure Hydrocephalus displaying significantly enlarged ventricles. In each of our two example problems, we achieve a speedup of more 750 times compared to the full order problem, while introducing a comparably small additional system assembly overhead. The reduced solutions recover the full order solution with an error of less than 10% in most cases.

*Statement of significance*: In many fields, model order reduction is a key technique in enabling high-throughput numerical simulations, but remains largely unexploited for biomedical modeling of the brain. In this work, we introduce a novel technique for building reduced representations integrating simulations performed on other brain geometries derived from MRI. Using this technique, we may leverage a dataset of previous solutions to accelerate simulations on new geometries, making patient-specific modeling more feasible.

## Highlights

• A novel framework using image registration to map numerical solutions between brain geometries is proposed.
• A POD-based framework for model order reduction using snapshots from other brain geometries is introduced.
• The method is illustrated on two example problems typical of glymphatic modeling.
• We achieve a speedup of more than 750 times compared to the full order problem.
• The error on the reduced models are generally 10%, or less, compared to the full order problem.

## 1 Introduction

The fluid–structure interaction processes of the human brain has in recent years become an important research topic due to its relation to solute transport and brain clearance. In particular, the glymphatic system is a brain-wide perivascular transport route that provides for CSF-mediated solute and fluid transport within the brain. Adding to the discovery of the glymphatic system (Iliff et al., 2012), was the characterization of meningeal lymphatic vessels capable of draining CSF (Louveau et al., 2015, Aspelund et al., 2015). The CSF transport is crucial for brain health, and in particular its clearance of metabolic waste which seems to be facilitated during sleep (Xie et al., 2013, Bojarskaite et al., 2023, Eide et al., 2021). Reduced clearance is linked to the accumulation of toxic proteins such as amyloid-β and tau, which is the hallmark of Alzheimer’s disease (Hardy and Selkoe, 2002, Spillantini and Goedert, 2013), as well as Lewy-bodies and α-synuclein which has been tied to Parkinson’s disease especially (Goedert, 2001).

Multiple computational models have been proposed for assessing glymphatic clearance (Corti et al., 2024, Dreyer et al., 2024, Fumagalli et al., 2024, Hornkjøl et al., 2022, Johnson et al., 2023, Valnes et al., 2020, Vardakis et al., 2020, Vinje et al., 2023). Common for these studies is that only few subjects are studied as multi-physics processes of subject specific models are currently a formidable computational challenge. For instance, in Hornkjøl et al. (2022) around 30 000 CPU hours were used for a single subject considering the solute transfer and fluid flow of CSF in order to investigate the influence of CSF mediated solute transport on glymphatic function. Similarly, in Vinje et al. (2023) approximately 64 000 CPU hours were used to assess the influence of sleep and sleep deprivation in 24 subjects. As such, there is a need for computational approaches that enable larger cohorts of subjects.

Model order reduction (MOR) is a technique which aims to alleviate the computational cost of performing simulations by leveraging previous simulations (Chinesta et al., 2016, Hesthaven et al., 2016), often called snapshots. With our pipeline (Mardal et al., 2022), magnetic resonance images (MRIs) are segmented via FreeSurfer (Dale et al., 1999), meshed in SVMTK and exported to FEniCS (Logg et al., 2012) format with proper subdomain markers and adjustable resolution. A main issue, however, is the fact that the subject specific meshes will differ in terms of geometry, as well as the number of degrees of freedom. As such, a main challenge to enable MOR is to construct mappings that are flexible and robust with respect to inconsistencies between the meshes.

Due to the direct relationship between the underlying MRI and the computational mesh, we propose an approach to this challenge which relies on image-based registration methods. Image registration is an important tool in radiology, as it allows for more direct inter- and intra-subject comparisons of imaging data. Accordingly, many approaches have been proposed for brain imaging specifically (Fischer and Modersitzki, 2003, Andersson et al., 2008, Ashburner, 2007, Ashburner and Friston, 2011, Hoffmann et al., 2022, Postelnicu et al., 2009, Avants et al., 2008, Modat et al., 2014), see Klein et al., 2009, Klein et al., 2010 for a detailed comparison of some of these methods. In this work, we apply the image registration method introduced in Avants et al. (2008), available from the Advanced Normalization Tools (ANTs) image toolbox, to $T_{1}$-weighted brain MRI. We thus compute image-to-image mappings to act as a vehicle for inter-subject brain transformations which can be applied to meshes derived from MRI. This means that the inter-geometry transformation is completely independent of the construction of the mesh representing the brain geometry, but rather entirely tied to the underlying MRI.

Our approach is tested and implemented on a dataset of 101 $T_{1}$-weighted brain MRI of human patients. All patients in the dataset were imaged while under investigation for suspected CSF disorders, although not all subjects were found to have a defined CSF disorder or neurological disease. The main pathology present in this dataset is patients diagnosed with idiopathic Normal Pressure Hydrocephalus (iNPH), which is a combined CSF and neurodegenerative disease with overlap toward Alzheimer’s disease (Eide, 2025). In patients with iNPH, disturbances in CSF flow is associated with enlarged ventricles (Malm and Eklund, 2006) and thus fundamentally changes the geometry of the brain. As such, we compare the performance of the method on an iNPH patient with another patient from a group of reference (REF) patients. After thorough diagnostic assessment, the REF individuals had no identified CSF disturbance or other neurological disease, and were therefore considered as close to healthy. The method is illustrated on two example problems typical of numerical modeling of glymphatic function, which we employ illustrate the ability of the method to recover the full order solutions as well as computational costs involved in the method.

**Fig. 1:**
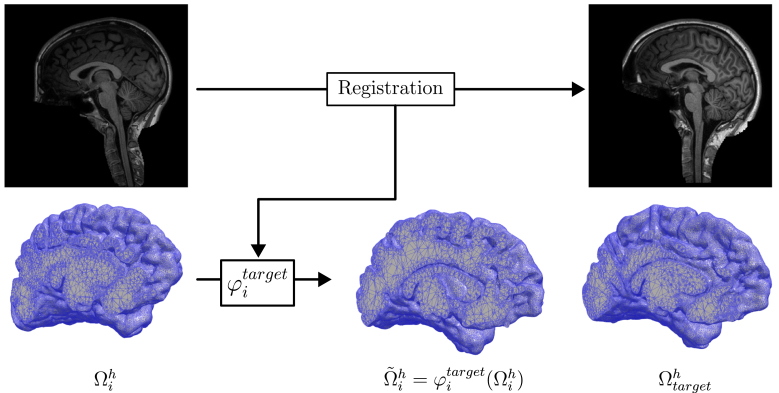
Image registration applied to meshes: Registration is performed by matching the MRI $I_{i}$ (top left) to $I_{target}$ (bottom left), thus obtaining $\varphi_{i}^{target}$. Using meshing tools we derive volumetric meshes from MRI, and apply $\varphi_{i}^{target}$ to the mesh $\Omega_{i}^{h}$ (top) to obtain the deformed mesh $\varphi_{i}^{target}\left(\Omega_{i}^{h}\right)$ (middle right), which approximates the target mesh $\Omega_{target}^{h}$ (bottom right).

## 2 Geometric model order reduction

### 2.1 Image registration

The goal of image registration is to compute a mapping from one image to another, by matching $T_{1}$-weighted MRI signal intensities $I_{i}$ to a target image $I_{target}$. We consider voxelized images on a 256 × 256 × 256 grid, with a uniform 1mm resolution. Through optimization, a mapping $\varphi_{i}^{target}$ from image $I_{i}$ to $I_{target}$ is computed such that $I_{i}\circ \varphi_{i}^{target}\approx I_{target}$. The concept of applying the deformation $\varphi_{i}^{target}$ obtained by image registration to a finite element mesh rests on the fundamental correspondence between the MRI of a brain $I_{i}$, represented by an image, and any associated mesh $\Omega_{i}^{h}\subset R^{3}$, represented by a set of points. Brain surface meshes are obtained through an iterative process of approximating surface features from a segmentation of the gray cortical matter and ventricles, and accordingly the mesh of each brain is unique. As these meshes describe complex surfaces with significant curvature, they are highly unstructured, and we do not expect the points of the mesh to correspond exactly with the voxels of the image. Depending on the mesh parameter h, the finite element mesh may also be locally far more dense or far less dense than the image. The critical advantage of using images for registration rather than meshes is that every image is defined on the same grid, while mesh nodes will not necessarily correspond.

Consequently, when $\varphi_{i}^{target}$ is applied to $\Omega_{i}^{h}$, we do not recover $\Omega_{target}^{h}$, but rather an approximation of the target mesh. We refer to Fig. 1, which illustrates how the mesh i is mapped to the target mesh. We notice that the deformed mesh ${\overset{\sim }{\Omega }}_{i}^{h}≔\varphi_{i}^{target}\left(\Omega_{i}^{h}\right)$ outlines the same geometry as $\Omega_{target}^{h}$, however the two meshes are distinct as the mesh nodes do not correspond.

### 2.2 Model order reduction

**Fig. 2:**
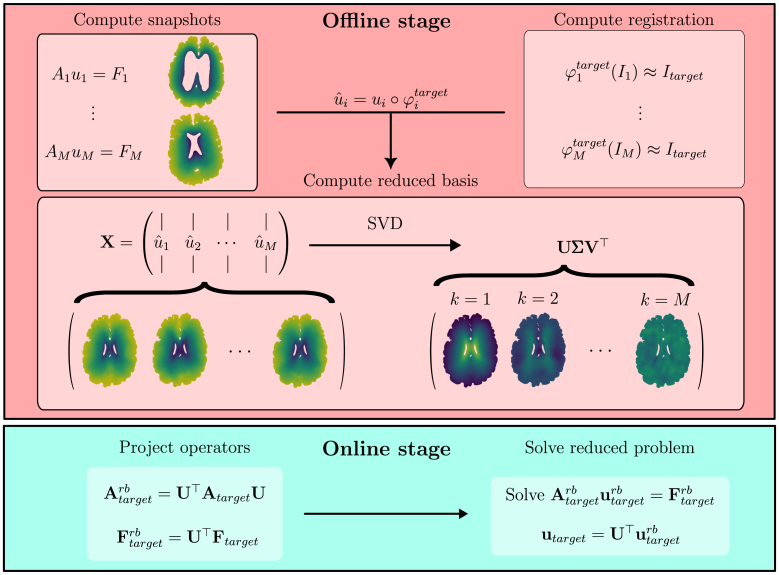
Schematic illustration of the reduced order approach. During the offline stage snapshot solutions on individual brain geometries and deformation fields from image registration are computed. Solutions are mapped to the target geometry, and a reduced basis is found by computing the SVD. We show three examples of SVD modes, obtained by computing the matrix U, to illustrate the reduced basis. During the online phase the reduced basis is used to project solution operators into a reduced space, enabling faster forward solves.

We apply an offline-online approach to model order reduction based on the precomputing of a set of high-fidelity snapshot solutions (Sirovich, 1991). We consider a situation where many images $I_{i}$ and associated meshes $\Omega_{i}^{h}$ are available, allowing us to precompute solutions on these geometries during the offline phase. For any given problem and associated parameters, this process only needs to be done once. During the online phase, we consider a situation in which we want to solve the problem of interest on a new, unseen target brain geometry $\Omega_{target}^{h}$. Based on the image representation of this brain geometry, we compute the mapping of each brain in the dataset to the target geometry. Using this dataset of previous solutions, we may thus compile a set of snapshot solutions on the geometry of interest, and compute a low-dimensional basis representation using the Proper Orthogonal Decomposition (POD) (Volkwein, 2011). During the online phase, the same underlying problem is solved in the reduced basis representation, significantly reducing the computational cost of the forward problem. In Fig. 2, we illustrate the steps involved in our model order reduction approach.

#### 2.2.1 Precomputing snapshots

Consider a general variational problem, with a bilinear form a(⋅,⋅), defined on $V_{i}\subseteq H\left(\Omega_{i}\right)$, where H is a Hilbert space for a geometry $\Omega_{i}\subset R^{3}$. Assume that a(⋅,⋅) is continuous and coercive on $V_{i}$. Let $u_{i}\in V_{i}$ be such that:

(1) $$a_{i}\left(u_{i},v_{i}\right)=f_{i}\left(v_{i}\right)\;\forall v_{i}\in V_{i}$$

Where $f_{i}\left(\cdot \right)$ is the linear form of the PDE. This problem can be solved numerically by introducing a discretization of non-degenerate triangulations $\Omega_{i}^{h}$ of $\Omega_{i}$, which we refer to as a mesh. Different meshes will have different numbers of degrees of freedom, and a direct mapping between meshes will, in general, be degenerate. Consequently, the function spaces $V_{i}$ also have a different number of degrees of freedom and cannot be mapped directly to each other. From each mesh, we can build a conforming subspace $V_{i}^{h}\subseteq V_{i}$ and solve the discrete variational problem of finding $u_{i}^{h}\in V_{i}^{h}$ such that:

(2) $$a_{i}\left(u_{i}^{h},v_{i}\right)=f_{i}\left(v_{i}\right)\;\forall v_{i}\in V_{i}^{h}$$

The discrete problem can then be solved for each geometry $\Omega_{i}^{h}$ and generating a dataset of solutions on M available geometries:

$${\left\{u_{i}^{h}\in V_{i}\left(\Omega_{i}^{h}\right)\right\}}_{i=1}^{M}$$

We aim to map these solutions to the target geometry $\Omega_{target}^{h}$. For each image-mesh pair in the dataset, we thus compute the mapping $\varphi_{i}^{target}$, based on the MRI $I_{i}$ and $I_{target}$, and subsequently apply the transformation to each precomputed solution $u_{i}$.

This procedure yields a set of solutions on the deformed geometries ${\overset{\sim }{\Omega }}_{i}^{h}$ which approximate the target geometry $\Omega_{target}^{h}$:

$$\left\{{\overset{\sim }{u}}_{i}^{h}=u_{i}^{h}\circ \varphi_{i}^{target}\right\}$$

A key observation is that the deformed solutions ${\overset{\sim }{u}}_{i}^{h}$ will not be elements of $V_{i}\left({\overset{\sim }{\Omega }}_{i}^{h}\right)$. The change of variables means that Eq. (2) will generally not be satisfied on ${\overset{\sim }{\Omega }}_{i}^{h}$. In order for the variational form to be satisfied on this geometry, we need to modify the differential operators to be consistent with $\varphi_{i}^{target}$, i.e set:

(3) $${\overset{ˆ}{\nabla }}_{i}=J_{\varphi_{i}^{target}}\nabla_{i}\;\text{and}\;\text{d}{\overset{\sim }{\Omega }}_{i}^{h}=\left|J_{\varphi_{i}^{target}}\right|\text{d}\Omega_{i}^{h}$$

Where $J_{\varphi_{i}^{target}}$ denotes the Jacobian of $\varphi_{i}^{target}$ in each point $x\in \Omega_{i}^{h}$. By defining modified bilinear and linear forms ${\overset{ˆ}{a}}_{i}\left(\cdot ,\cdot \right)$ and ${\overset{ˆ}{f}}_{i}$ using these differential operators, as well as a modified function space ${\overset{ˆ}{V}}_{i}\left({\overset{\sim }{\Omega }}_{i}^{h}\right)$ each deformed solution ${\overset{\sim }{u}}_{i}^{h}$ will satisfy:

(4) $${\overset{ˆ}{a}}_{i}\left({\overset{\sim }{u}}_{i}^{h},{\overset{\sim }{v}}_{i}\right)={\overset{ˆ}{f}}_{i}\left({\overset{\sim }{v}}_{i}\right)\;\forall {\overset{\sim }{v}}_{i}\in {\overset{ˆ}{V}}_{i}\left({\overset{\sim }{\Omega }}_{i}^{h}\right)$$

The act of sampling solutions from various geometries, is therefore essentially equivalent to sampling solutions with modified differential operators.

However, sampling solutions from Eq. (4) is itself not enough. As seen in Fig. 1, while the mesh ${\overset{\sim }{\Omega }}_{i}^{h}$ is a reasonable approximation of $\Omega_{target}$, the cells and nodes of the mesh will still be distinct from $\Omega_{target}^{h}$, which is where $u_{target}^{h}$ is defined. We must therefore additionally extend each solution ${\overset{\sim }{u}}_{i}^{h}$ from ${\overset{\sim }{\Omega }}_{i}^{h}$ to $\Omega_{target}^{h}$. For nodes where the two meshes overlap, this can be directly done through interpolation of the finite element basis. At the boundary, some nodes in ${\overset{\sim }{\Omega }}_{i}^{h}$ may not lie within a cell of $\Omega_{target}$ due to the registration not being exact. In such cases, we perform extrapolation by gaussian weighting of the closest neighboring cell values. We use the notation ${\overset{ˆ}{u}}_{i}^{h}$ to denote the extension of the deformed solution ${\overset{\sim }{u}}_{i}^{h}$ from ${\overset{\sim }{\Omega }}_{i}^{h}$ to $\Omega_{target}^{h}$.

#### 2.2.2 Constructing the reduced basis

Once the dataset of snapshots on the target geometry $\Omega_{target}^{h}$ has been computed, we aim to use these solutions to solve the underlying problem in a reduced space. In particular, we aim to replace $V_{target}\left(\Omega_{target}^{h}\right)$ by the space spanned by these sample solutions ${\overset{ˆ}{V}}_{M}=\text{span}\left\{{\overset{ˆ}{u}}_{1}\ldots {\overset{ˆ}{u}}_{M}\right\}$ to solve Eq. (2). The purpose of the POD is to construct an optimal orthonormal basis for this space.

We compute this basis by the following approach: Each snapshot has D degrees of freedom, given by the mesh $\Omega_{target}^{h}$ and the order of the finite element space of choice. When each solution contains n fields, with D degrees of freedom, we treat each field as independent and consider the solution to contain $\overset{\sim }{D}=n\cdot D$ degrees of freedom. We express each solution as a vector with $\overset{\sim }{D}$ components and stack them column-wise to construct a snapshot matrix $X\in R^{\overset{\sim }{D}\times M}$:

$$X=\left(\begin{array}{cccc} | & | & | & |\\ {\overset{ˆ}{u}}_{1} & {\overset{ˆ}{u}}_{2} & \cdots & {\overset{ˆ}{u}}_{M}\\ | & | & | & | \end{array}\right)$$

Computing a low-dimensional representation of this data using the POD corresponds to finding the d-rank matrix U, such that:

(5) $$U=\underset{U\in R^{\overset{\sim }{D}\times d}}{\mathrm{argmin}}{\left\|X-UU^{⊤}X\right\|}_{F}\;U^{⊤}U=I$$

Where $I\in R^{d\times d}$ denotes the d×d identity matrix. By computing such a matrix U with rank $d\ll \overset{\sim }{D}$, we can obtain a very low-dimensional orthogonal basis of the space spanned by X. From the Eckart–Young theorem (Eckart and Young, 1936), the optimal solution to (5) is to compute the SVD of the matrix:

$$X=\overset{ˆ}{U}\Sigma {\overset{ˆ}{V}}^{⊤}$$

The optimal d-rank basis representation of X can be found by extracting the first d columns of the left-side singular matrix, setting $U={\overset{ˆ}{U}}_{1:d}$. The matrix $\Sigma \in R^{S\times M}$, $S=\min\left(\overset{\sim }{D},M\right)$ is the singular value matrix, which consists entirely of 0’s, except for the diagonal of the upper S×S sub-matrix which contains the singular values ${\left\{\sigma_{i}\right\}}_{i=1}^{S}$. The singular values are key to understanding the POD as they measure the error of the d-rank approximation of X (Eckart and Young, 1936):

$${\left\|X-UU^{⊤}X\right\|}_{F}=\overset{S}{\underset{i=d+1}{\sum }}\sigma_{i}$$

Having obtained U, we subsequently seek to solve (2) in the low-dimensional space spanned by this basis. This involves finding $u_{target}^{rb}\in V_{target}^{rb}$, where $V_{target}^{rb}$ is defined as:

$$V_{target}^{rb}=\text{span}\left\{U_{1}\ldots U_{d}\right\}$$

We then subsequently solve the reduced variational problem:

(6) $$a_{target}\left(u_{target}^{rb},v_{target}\right)=f_{target}\left(v_{target}\right)\;\forall v_{target}\in V_{target}^{rb}$$

In practice, we can obtain (6) from (2) by projection:

$$A_{target}^{rb}=U^{⊤}A_{target}U\;{\left(A_{target}^{rb}\right)}_{i,j}=a_{target}\left(U_{i},U_{j}\right)$$

$$F_{target}^{rb}=U^{⊤}F_{target}\;{\left(F_{target}^{rb}\right)}_{i}=f_{target}\left(U_{i}\right)$$

The reduced basis solution $u_{target}^{rb}$ can now be obtained by solving $A_{target}^{rb}u_{target}^{rb}=F_{target}$. The high-dimensional representation can then be recovered by inverse projection $u_{target}=Uu_{target}^{rb}$.

## 3 Models and data

### 3.1 Models

#### 3.1.1 Two-compartment model of tracer distribution

As a first example, we implement the model proposed in Riseth et al. (2023), at steady state. This is a diffusion-dispersion continuum model, simulating exchange of MRI tracer concentration between the extracellular space (ECS) and perivascular space (PVS) surrounding blood vessels in the brain, as well as some clearance into blood from perivascular spaces. The model assumes that the tracer concentration in blood and advective transport is negligible, and that tracer molecules may only disperse from the PVS into blood, but not directly from the ECS. Concentration at the fluid filled ventricles and subarachnoid spaces (SAS) surrounding the brain are modeled as Robin boundary conditions. We implement the model at steady-state, estimating the peak in concentration, normally seen 15−20h after injection. The model is formulated as the following set of differential equations:

(7) $$-\nabla \cdot \left(n_{e}D_{e}\nabla c_{e}\right)=\pi_{ep}\left(c_{p}-c_{e}\right)\text{in}\Omega -\nabla \cdot \left(n_{p}D_{p}\nabla c_{p}\right)=-\pi_{ep}\left(c_{p}-c_{e}\right)-\pi_{pb}c_{p}\text{in}\Omega -n_{e}D_{e}\nabla c_{e}\cdot n=k_{e}\left(c_{e}-c_{CSF}^{\alpha }\right)\text{on}\partial \Omega_{\alpha }-n_{p}D_{p}\nabla c_{p}\cdot n=k_{p}\left(c_{p}-c_{CSF}^{\alpha }\right)\text{on}\partial \Omega_{\alpha }$$

Where $c_{e}$ and $c_{p}$ denote the concentration in the extracellular and perivascular spaces, respectively. A complete list of model parameters and values are listed in Table 1. The quantity $c_{CSF}^{\alpha }$ is the concentration at the boundary between tissue and CSF. We consider an idealized case of tracer concentration in the CSF, which we estimate as (Riseth et al., 2023):

$$c_{CSF}^{\alpha }=a_{\alpha }\phi^{-1}\left(-e^{-t/\tau_{1}}+e^{-t/\tau_{2}}\right)$$

Where α∈{ventricle, pial}, with $a_{\text{pial}}=0.52\;{\mathrm{mm}}^{2}$/s and $a_{\text{ventricle}}=0.2\;{\mathrm{mm}}^{2}$/s, ϕ=0.2 is the volume fraction, $\tau_{1}=4.43\cdot 10^{4}$ s and $\tau_{2}=8.5\cdot 10^{4}$ s. We set t=16.7h, which is the point where the estimated SAS concentration reaches its peak. The boundary condition models the influx of tracer from CSF spaces across a membrane at the ventricle and pial surfaces, as a Robin boundary condition. Each boundary surface concentration $c_{CSF}^{\alpha }$ is applied to the associated surface $\partial \Omega_{\alpha }$ denoting the interface between the parenchyma and the ventricles or SAS. We use the same surface conductivity for the ventricle and pial surfaces.

**Table 1:** Model parameters for Eq. (7), using ranges from (Riseth et al., 2023). We consider a variation of the model with a relatively low rate of clearance to blood.

Quantity	Description	Value	Unit
$n_{e}$	ECS volume fraction	0.2	–
$n_{p}$	PVS volume fraction	$0.2\cdot 10^{-1}$	–
$D_{e}$	ECS diffusion coefficient	$1.3\cdot 10^{-4}$	mm${}^{2}$/s
$D_{p}$	PVS diffusion coefficient	$3.9\cdot 10^{-4}$	mm${}^{2}$/s
$\pi_{ep}$	Transfer coefficient ECS-PVS	$2.9\cdot 10^{-2}$	1/s
$\pi_{pb}$	Transfer coefficient PVS-blood	$2.0\cdot 10^{-8}$	1/s
$k_{e}$	ECS surface conductivity	$1.0\cdot 10^{-5}$	mm/s
$k_{p}$	PVS surface conductivity	$3.7\cdot 10^{-4}$	mm/s

#### 3.1.2 Multi-compartment poro-elastic model (MPET)

As a second example, we implement a 7-compartment poro-elastic model (MPET) described in Dreyer et al. (2024), which is a modified version of the model in Tully and Ventikos (2011). A mathematical analysis of a generalized version of the model is provided in Lee et al. (2019). This model aims to simulate flow resistance in the brain, and the multi-compartment interactions are relevant for general glymphatic modeling. At steady state, ignoring elastic deformations, the governing equations are given by:

(8) $$-\nabla \cdot \frac{\kappa_{i}}{\mu_{i}}\nabla p_{i}=\underset{i\neq j}{\sum }\omega_{ij}\left(p_{i}-p_{j}\right)$$

Where $\kappa_{i}$ and $\mu_{i}$ model the fluid viscosity and permeability in compartment i and $\omega_{ij}$ models the fluid transfer coefficient between compartment i and j, following Tully and Ventikos, 2011, Guo et al., 2019. We refer to Dreyer et al. (2024) for a detailed explanation of each physical quantity, and considerations on relevant ranges estimated in the literature. In this work, we use the base variant described in Dreyer et al. (2024), and we summarize the model parameters in Table 2. We model the pressure in the capillary ($p_{c}$), arterial ($p_{a}$) and venous ($p_{v}$) blood compartments, as well as the perivascular spaces ($p_{pc},p_{pa},p_{pv}$) associated with each compartment, and the extracellular space ($p_{e}$).

In the pre-infusion phase the, boundary conditions associated with Eq. (8) are given by:

(9) $$\kappa_{a}\nabla p_{a}\cdot n=Q_{in}\;x\in \partial \Omega_{pial}$$

$$\kappa_{c}\nabla p_{c}\cdot n=-Q_{prod}\;x\in \partial \Omega_{ventricle}$$

$$\kappa_{v}\nabla p_{v}\cdot n=\beta_{1}\left(\frac{p_{DS}+p_{CSF}}{2}-p_{v}\right)\;x\in \partial \Omega_{pial}$$

$$\kappa_{pa}\nabla p_{pa}\cdot n=\beta_{2}\left(p_{CSF}-p_{pa}\right)\;x\in \partial \Omega_{pial}$$

$$\kappa_{pv}\nabla p_{pv}\cdot n=\beta_{3}\left(\frac{p_{DS}+p_{CSF}}{2}-p_{pv}\right)\;x\in \partial \Omega_{pial}$$

Where $Q_{in}$ models CSF flow rates due to arterial infusion from arteries penetrating the pial surface. At the ventricle surface there are no such penetrating arteries, and $Q_{prod}$ estimates flow rates across the ependymal cells of the ventricle surfaces modeled as CSF production in the choroid plexus. We consider only the stage prior to infusion testing modeled in Dreyer et al. (2024) and use $Q_{in}=B_{in}/\int \text{d}\partial \Omega_{pial}$, where $B_{in}$ is the arterial flow-rate, relating the transfer coefficient between blood vessels to the expected pressure drop (Dreyer et al., 2024). The flow rate in the choroid plexus is set to $Q_{prod}=0.33\;\text{ml/min}$, and we use $p_{DS}=8.4\;\text{mmHg}$, $\beta_{1}=\beta_{2}=10^{-3}$ and $\beta_{3}=10^{-7}$. In the remaining compartments, we set $\nabla p_{i}\cdot n=0$ on the SAS and ventricular surfaces.

**Table 2:** Model parameters for Eq. (8), as listed in the base model in Dreyer et al. (2024). .

Transfer coefficient	Value [Pa^−1^s^−1^]	Permeability	Value [m${}^{2}$]
$\omega_{a,c}$	$1.45\cdot 10^{-6}$	$\kappa_{a}$	$3.63\cdot 10^{-14}$
$\omega_{c,v}$	$8.75\cdot 10^{-6}$	$\kappa_{c}$	$1.44\cdot 10^{-15}$
$\omega_{c,pc}$	$8.48\cdot 10^{-10}$	$\kappa_{v}$	$1.13\cdot 10^{-12}$
$\omega_{pa,e}$	$1.86\cdot 10^{-7}$	$\kappa_{e}$	$2\cdot 10^{-17}$
$\omega_{pv,e}$	$1.65\cdot 10^{-7}$	$\kappa_{pa}$	$3\cdot 10^{-17}$
$\omega_{pa,pc}$	$10^{-6}$	$\kappa_{pc}$	$1.44\cdot 10^{-15}$
$\omega_{pc,pv}$	$10^{-6}$	$\kappa_{pv}$	$1.95\cdot 10^{-14}$
$\omega_{pc,e}$	$10^{-10}$		

### 3.2 Data and acquisition

In this work, we will apply the aforementioned registration techniques to a dataset of 101 $T_{1}$-weighted brain MRI. Parts of this dataset has been reported in previous works on glymphatic solute transport assessed with MRI (Ringstad et al., 2017, Ringstad et al., 2018). Participants in the study were all recruited while under clinical work-up for various CSF disorders, and imaged at the Department of Neurosurgery, University Hospital of Oslo-Rikshospitalet according to the protocol described in Ringstad et al. (2018). Study subjects underwent intrathecal gadobutrol injection and were imaged using MRI before injection, in addition to several time-points following injection. In this study, we only consider MRI acquired before intrathecal injection. Data examined in this study was collected in the period 2015–2016 and approved by the Regional Committee for Medical and Health Research Ethics (REK) of Health Region South-East, Norway (2015/96), the Institutional Review Board of Oslo University Hospital (2015/1868) and the National Medicines Agency (15/04932-7). The study was conducted following the ethical standards of the Declaration of Helsinki of 1975 (revised in 1983). Study participants were included after written and oral informed consent. No additional data was collected for this work.

In Table 3, we detail the patient groups present in the dataset. The largest single patient group are subjects diagnosed with iNPH, which is a neurodegenerative disease with symptoms like urinary incontinence, gait disturbance and dementia (Eide, 2025, Malm and Eklund, 2006). The iNPH diagnosis is based on the American-European guidelines (Relkin et al., 2005), which not only consider the ventriculomegaly, however a hallmark of iNPH is enlarged cerebral ventricles associated with CSF flow disturbances (Ringstad et al., 2017, Eide et al., 2020). This cohort is therefore expected to display the most consistent geometric variations compared to other patient groups. In this work, we only consider the effect of brain geometry on simulations of glymphatic function and accordingly consider this group as distinct. The remaining 70 patients in the dataset consist of three main groups: (1) REF refers to patients who where not diagnosed with a CSF-related disorder following clinical work-up. These individuals may be considered as being close to healthy. (2) Cysts, refers to patients diagnosed with pineal or arachnoid cysts. (3) Other, refers to the remaining 26 patients who are diagnosed with other CSF disorders such as Spontaneous Intracranial Hypotension (SIH) or Idiopathic Intracranial Hypertension (IIH). The non-iNPH cohort is therefore highly heterogeneous, however, from a purely geometrical perspective this group will, on the whole, be geometrically distinct from the iNPH group.

**Table 3:** Characteristic data for subject groups in this study. Other refers to patients diagnosed with other CSF disorders, such as Spontaneous Intracranial Hypotension (SIH), Idiopathic Intracranial Hypertension (IIH).

Group	N	Age	Sex (M/F)	Height [cm]	Weight [kg]
iNPH	31	(71 ± 7)[46 − 80]	23/8	(176 ± 9)	(83 ± 17)
Non-iNPH	70	(40 ± 13)[19 − 72]	18/52	(169 ± 21)	(79 ± 20)
REF	18	(35±11)[22−64]	4/14	(163±41)	(75±25)
Cysts (pineal, arachnoid)	26	(39±13)[19−68]	6/20	(172±8)	(81±12)
Other	26	(43±14)[24−72]	8/18	(171±8)	(79±22)

Total	101	(49 ± 18)[19 − 80]	41/60	(171 ± 19)	(81 ± 25)

### 3.3 Implementation details

For each MRI in the dataset, we build 3D digital meshes using the pipeline described in Mardal et al. (2022). First, we perform a FreeSurfer segmentation (Dale et al., 1999) of the brain, which allows us to extract the pial and ventricular surfaces. Volumetric meshes are created using SVMTK (Valnes and Schreiner, 2021), generally consisting of on the order of $10^{5}$ nodes. Simulations are performed using finite element schemes in dolfinx (v.0.9.0) (Baratta et al., 2023). For the two compartment model we use a second order Lagrange basis for the trial and test spaces, and for the MPET model we use first order Lagrange elements. In each case, this leads to a number of degrees of freedom on the order of $1.5\cdot 10^{6}$ for the high-fidelity two-compartment model and $0.8\cdot 10^{6}$ for the MPET model, with slight variations depending on the specific brain geometry. Image registration is performed using the framework introduced in Avants et al. (2008), implemented in the Advanced Normalization Tools (ANTS) toolbox (v.2.5.4)

For computational purposes, we consider only the cortical surface of the brain and the third and lateral ventricles. Segmentations of the fourth ventricle are generally unreliable when building finite element meshes because the channel from the fourth to the third ventricle will be only a few voxels wide, or disappear entirely. In the interest of time, we therefore choose to remove the cerebellum, as well as the fourth ventricle and the brain stem from the model to remove the need for manual correction of the segmentation.

**Fig. 3:**
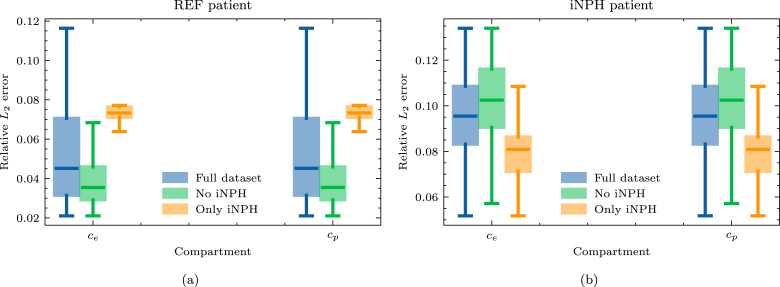
Relative error in the $L_{2}$ norm on different subsets of the full dataset. We show results for (a) a REF patient and (b) a patient diagnosed with iNPH.

## 4 Results

In the following, we implement the approach outlined in Section 2, and apply it to the two models from Section 3.1. We choose two target geometries: One patient in the REF group, with no known CSF disorder or neurological disease, and another patient diagnosed with iNPH displaying ventriculomegaly. The REF patient meanwhile displays ventricular morphology within a normal range and we therefore consider the brain of this patient as a close approximation of a healthy brain. For each target subject, we measure the average approximation error incurred by mapping solutions from another geometry, as well as the singular values and reconstruction error due to the reduced approximation in terms of the relative $L_{2}$ error. In each case, we investigate the effect of considering the entire dataset, as well as when considering only the brains of iNPH or non-iNPH patients. In this regard, we consider the non-iNPH patient brains as representative of typical brain geometric variations, and the iNPH patient brains as representative of a class of brain geometries with enlarged ventricles. Finally, we show speedup results when using the reduced method and estimate the computational overhead associated with our approach.

### 4.1 Two-compartment model of tracer distribution

#### 4.1.1 Approximation by any other geometry

The relative error on the ECS and PVS concentrations due to mapping solutions from one brain geometry to another appears to depend strongly on the target patient group when applied to Eq. (7), as seen in Fig. 3. For the patient in the REF group, in Fig. 3(a), the error is lower when considering only the brains of patients who are not diagnosed with iNPH. A similar, but opposite trend can be observed for the iNPH patient target brain, as seen in Fig. 3(b). On this target geometry, the relative error is also consistently larger across all dataset subdivisions.

#### 4.1.2 Reduced order modeling

**Fig. 4:**
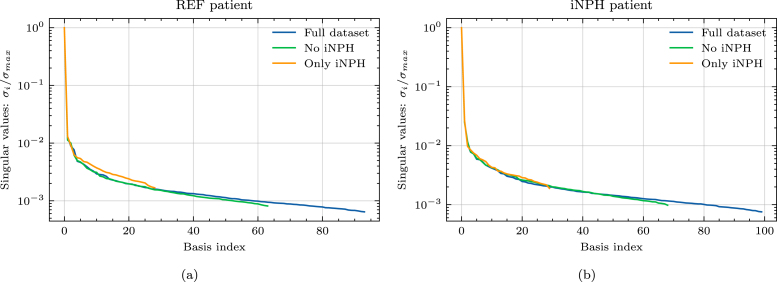
Normalized singular values as a function of the basis size. We show results for (a) a REF patient and (b) a patient diagnosed with iNPH.

**Fig. 5:**
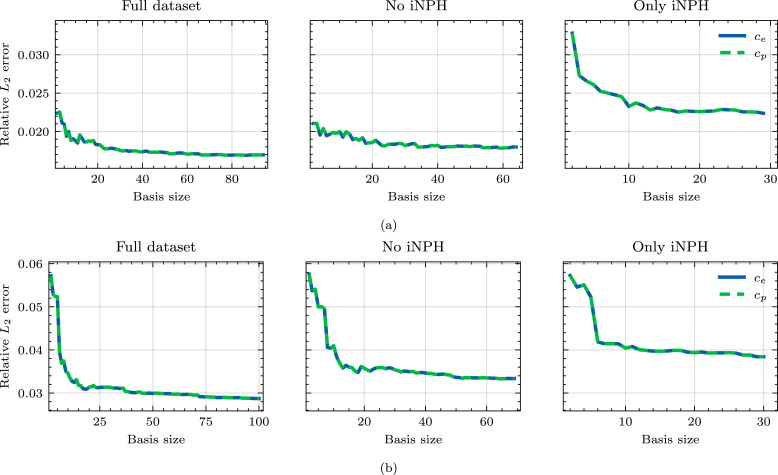
Relative error in the $L_{2}$ norm of the reduced solution compared to the high-fidelity solution. We show results for (a) a REF patient and (b) a patient diagnosed with iNPH.

On both target brain geometries, the singular values appear to decay by 3 orders of magnitude from the first singular value, to the size of the dataset, as seen Fig. 4. This decay is consistent across dataset subdivisions. Neither the REF, nor the iNPH patient display any clear advantage due to including only brains from the same patient group. This trend is further confirmed by Fig. 5, where using the full dataset leads to lower error on both target brains. The minor exception to this rule is the middle plot of Fig. 5(a), where we notice that on the REF patient brain, excluding the patients diagnosed with iNPH appears to yield comparable results to using the entire dataset.

**Fig. 6:**
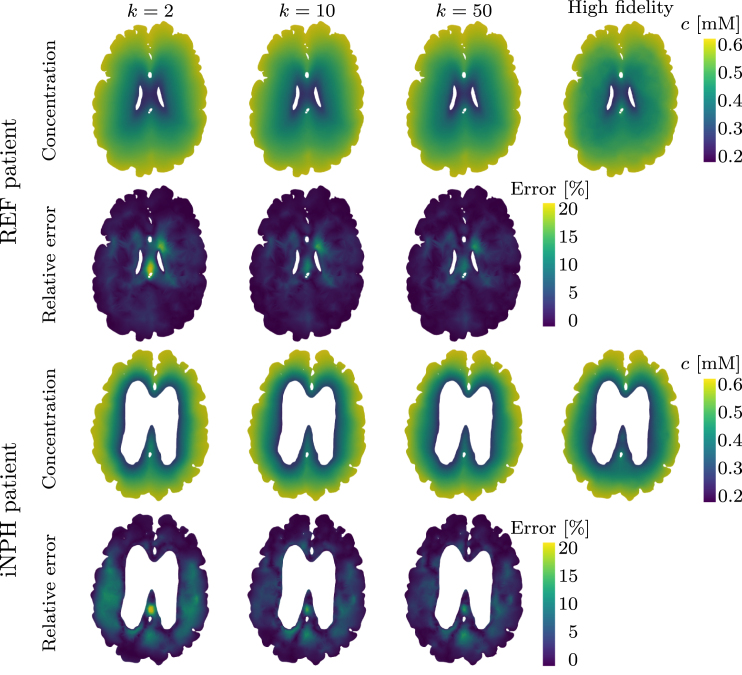
Comparison of concentration in the extracellular compartment $c_{e}$ using k=2,10,50 bases in the reduced solution for both the REF patient and the iNPH patient. We plot the high-fidelity solution for reference, and display the relative $L_{2}$ error of the reduced solutions compared to the full problem. The colorbar for the relative error is capped at 20% for illustrative purposes.

In Fig. 6, we plot an example of an axial slice showing the ECS concentration computed from three reduced models with different numbers of bases k, compared to the high-fidelity model. We notice that even in the k=2 case, the reduced solution is generally very similar to the high-fidelity solution both for the REF patient brain and the iNPH patient brain.

**Fig. 7:**
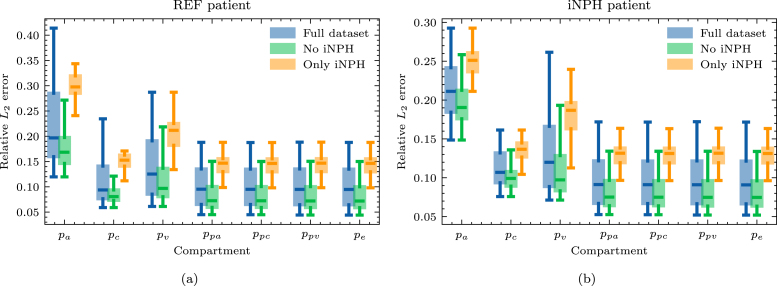
Relative error in the $L_{2}$ norm on different subset of the full dataset. We show results for (a) a REF patient and (b) a patient diagnosed with iNPH.

### 4.2 Multi-compartment poro-elastic model (MPET)

#### 4.2.1 Approximation by any other geometry

When solving Eq. (8), there does not appear to be any advantage gained from only mapping solutions from the same subject group, as shown in Fig. 7 where we plot the relative $L_{2}$ error on the pressure in the 7 compartments. In particular, the relative error is larger when mapping solutions from brains of patients with iNPH in all compartments and on both target geometries. Indeed, in all cases, the relative error appears to be lower when considering only the brains of patients in the dataset who are not diagnosed with iNPH.

#### 4.2.2 Reduced order modeling

**Fig. 8:**
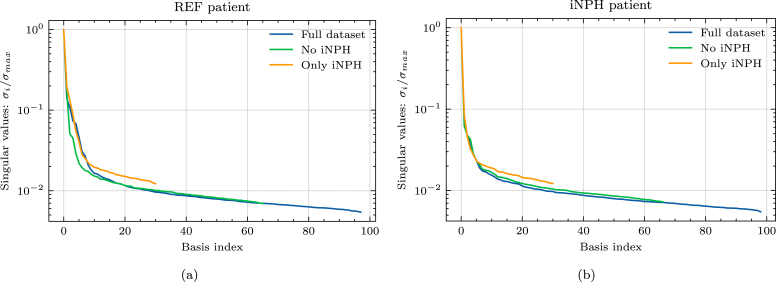
Normalized singular values as a function of the basis size. We show results for (a) a REF patient and (b) a patient diagnosed with iNPH.

On the MPET problem, the singular values appear to decay by 2 orders of magnitude compared to the largest singular value when using the entire dataset of brain geometries, as seen in Fig. 8. We also notice that the rate of decay on both target brains is slightly slower when using only iNPH brains, thus reflecting the results in Fig. 7. Overall, there are no significant differences between the singular value spectra of the REF and iNPH target patients.

**Fig. 9:**
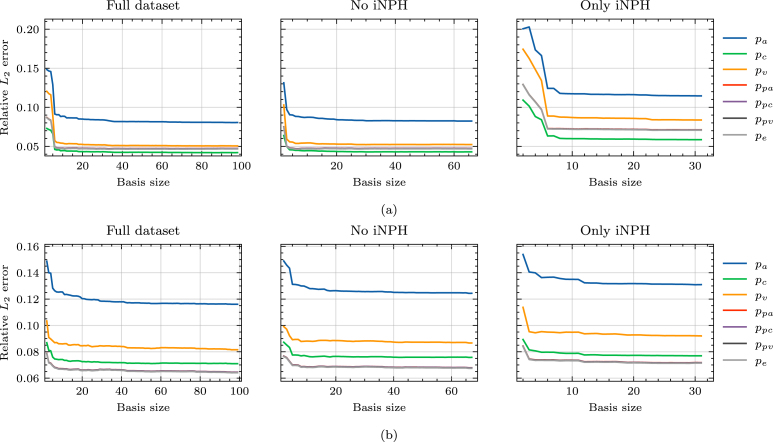
Relative error in the $L_{2}$ norm of the reduced solution compared to the high-fidelity solution. We show results for (a) a REF patient and (b) a patient diagnosed with iNPH.

**Fig. 10:**
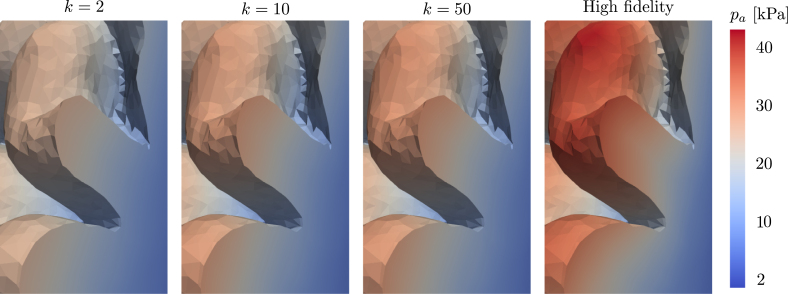
Comparison of pressure profile in a cortical fold in the arterial compartment $p_{a}$ using k=2,10 and 50 bases compared to the high-fidelity solution. We show a zoom on a sagittal slice of a cortical fold in the left hemisphere of the REF target patient.

The relative error due to the reduced basis approximation, shown in Fig. 9, on the MPET problem follows a similar trend as the two-compartment problem in Fig. 5. In particular, we notice that on the REF target patient, seen in Fig. 9(a), there is effectively only a minor difference in performance between using the entire dataset (left) and when considering only the non-iNPH patients (middle). Only including brains from iNPH patients in the snapshot matrix leads to significantly larger errors on all compartments. We notice a similar trend on the iNPH target patient, in Fig. 9(b), although the gaps between the dataset partitions appear less significant. In this case, it appears advantageous to use the entire dataset, and thus include the brains of patients with iNPH. The results in Fig. 9 also display significant differences in the errors on the various compartments in the MPET model. In all cases, the arterial compartment is the clear outlier, leading to significantly larger errors than other fields. For this particular field, the boundary condition leads to a build-up of pressure around cortical folds. Fig. 10 shows an example of how the pressure profile in the high-fidelity solution depends strongly on the shape of any given fold. The reduced solutions are not able to fully recover the sharp pressure gradient in the high-fidelity problem.

### 4.3 Computational costs and speedup

**Fig. 11:**
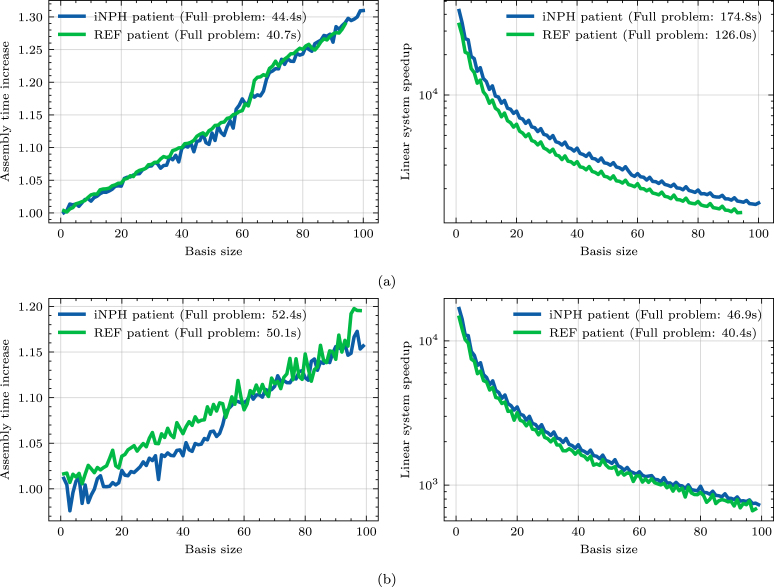
Speedup of the forward solve compared to the full problem and increase in the assembly time when using the reduced model when applied to (a) the two-compartment problem Eq. (7) and (b) the MPET problem Eq. (8). We use reduced bases computed from the full dataset for example purposes.

The main goal of reduced order modeling is to reduce computational cost by decreasing the dimensionality of the problem by replacing Eq. (1) with Eq. (6). However, as illustrated in Fig. 2, this requires projecting the full space operators into the reduced space, thereby creating an additional system assembly overhead. In Fig. 11 we quantify these two aspects of the online computational cost of the reduced model on the two example problems, using the entire dataset to generate the reduced basis in each case. The full and reduced problems are solved in serial on a Intel Xeon-Gold 6138 2.0 GHz/6230R 2.1 GHz CPU, we display the average time of five experiments, following compilation. Slight spikes in computational time is due to variations in resource availability on the cluster nodes used for running experiments. The left plots in Fig. 11, Fig. 11 show the increase of the assembly time at different basis sizes compared to the full problem. For the two-compartment model, the assembly time increases by 30% at the largest basis size, and 19% in the MPET model. The right plots in Fig. 11, Fig. 11 show the speedup due to solving the reduced linear system, i.e., the full problem solve time divided by the reduced solver time. For both problems, we obtain a speedup on the order $10^{4}$ for the smallest basis sizes, down to 1500 in two-compartment case and 750 in the MPET case for the largest problem sizes. In practical terms, the linear system solve time is thus reduced to less than 0.1 s in all cases. We note that the full problem solve times differ significantly between the two example problems due to differences in the number of degrees of freedom. Our approach also introduces a range of computational costs associated with the offline phase, including the creation of the snapshot solutions, image registration and computing the SVD. The most costly part of this process, by far, is the image registration step. On 8 processors using the aforementioned CPU, performing registration using the images in this dataset takes approximately 30 minutes, on average. However, this may vary importantly depending on the target patient and the resolution of the images that are used.

## 5 Discussion

### 5.1 Approximation by any other geometry

As a first step towards reduced order modeling, we evaluate the error of each solution, deformed from another geometry, in Fig. 3, Fig. 7. We notice that the errors are generally significantly higher in the 7-compartment pressure model, with median errors ranging from 8–20% when excluding iNPH patients on both target subjects. Meanwhile in the two-compartment tracer model, the median error when considering only patients from the same cohort is 4% on the REF target patient and 8% on the iNPH target patient. The arterial compartment of the 7-compartment model in Fig. 7 is especially difficult to approximate using solutions from other geometries, largely as a result of the boundary conditions. At the cortical surface, the arterial compartment has a pure Neumann condition on the pial surface, with a substantial influx due to $Q_{in}$. As the pial surface is a folded sheet with relatively tight folds, this causes an artificial build-up of pressure in small areas. The solution, therefore, depends too strongly on the shape of the cortical folds, which image registration is not able to consistently capture, leading to an median $L_{2}$ error of more than 20% in most cases. This effect is visible in the high-fidelity solution in Fig. 10. We also note that the capillary compartment has a similar boundary condition to the arteries, but on the ventricular, rather than the pial, surface. As seen in Fig. 7, the median error on this compartment is significantly lower, closer to 8%, as a result of the ventricular surface being more regular. Meanwhile, the boundary condition on the two-compartment problem leads to more smoothly varying solutions at the boundary, and as a result the errors in Fig. 3 are consistently lower than in Fig. 7.

The other notable impact of the boundary conditions is the importance of considering the diagnosis of each patient when mapping solutions between brains. It is notable that in Fig. 3, the relative error on the REF target patient is considerably lower when we only consider patients without iNPH, while on the iNPH patient the error is lower when we only consider patients with iNPH. In each case the median errors improve by close to 1 percentage point compared to using the entire dataset. However, this trend is not at all present in Fig. 7, where excluding the iNPH patients always leads to a lower relative error, generally lowering the error by more than 2 percentage points compared to using the entire dataset. We notice that in Eq. (7) the Robin boundary condition is placed on the ventricular and pial surfaces, while in the 7-compartment model all compartments, except the capillaries, have a homogeneous Neumann boundary condition on the ventricular surface. In the particular case of brains from patients with iNPH, the ventricular surface will inevitably be the most challenging aspect of image registration, as these anatomical features will vary considerably from the non-iNPH subjects. Consequently, applying the $c_{SAS}$ concentration on the ventricular surface in Eq. (7), means that matching this surface is critical to obtain a low error. In this case, it would therefore highly advantageous to use brains with the most similar morphology at the ventricles. Conversely, in the 7-compartment model, the homogeneous Neumann condition at the ventricles leads to a lower dependence in the particular surface morphology. In this case, the increased challenge of registering MRI of patients with iNPH likely outweighs any advantage that is gained by the ventricles being more similar. Indeed, in Fig. 7, we notice that including only the patients diagnosed with iNPH leads to a significantly larger increase in the relative error on the REF target patient than on the iNPH target patient.

### 5.2 Reduced order modeling

While Fig. 3, Fig. 7 show that there is some advantage to considering subsets of the full dataset when mapping solutions from one brain in the dataset to the target brain, this does not extend to reduced order modeling. For both problems, we build reduced bases based on the entire dataset, as well as when including only iNPH or non-iNPH patients. The results in Fig. 5, Fig. 9 show that using the entire dataset yields superior or equivalent results to removing subjects diagnosed with iNPH, and in all cases considering only the iNPH individuals is worse, even when the target does itself have iNPH. As such, these results indicate that it is generally better to use all available data when building a reduced basis, than to limit oneself to only patients within the same diagnosis group. The reduced order approach based on snapshots on previous solutions depends largely on the sampling of the relevant parameter space, which is in this case consists of other brain geometries. The most successful sampling strategy will therefore almost always be to employ a wide variety of different brain geometries when constructing the snapshot matrix. Our results indicate that it is more important to sample solutions from a maximum number of individuals, rather than to limit oneself to patients with the same pathology.

However, in the present dataset the number iNPH and non-iNPH patients are not equal, and the results in Fig. 5, Fig. 9 do not adjust for this imbalance. A distinct case may therefore be a situation where the number of available geometries on which one can compute snapshot solutions is limited to a fixed number. Would it be better to only consider patients with the same pathology in such a case? The decay of the singular values in Fig. 4, Fig. 8, largely answers this question. In Fig. 4(a), with a REF target patient, the iNPH only subset decays slightly slower than the full or non-iNPH options. Meanwhile, in Fig. 4(b) the three curves decay at essentially the same rate. In Fig. 8 the iNPH only subset shows the slowest decay in all cases. Accordingly, if we were to be able to add enough iNPH patients to equal the number of non-iNPH patients, we would still expect the error to decay more slowly when using only iNPH patients. More generally, it is likely often a better sampling strategy to not restrict the dataset to only patients with the same diagnosis. In any set of individuals, morphological variations will be significant and may therefore naturally provide a rich set of sample solutions. There may also be important differences between patients with the same diagnosis, and therefore relying only on this characteristic to decide which patients to include a priori, may ascribe greater precision to this marker than what is warranted for this application. Many pathologies will also display much less extreme morphological deformations than the iNPH condition, and thus in most cases it will likely be better to sample widely. We may also add that patients which represent the most extreme morphological deviations from the target patient will mostly lead to the image registration optimization method not converging, and these cases are thus effectively removed from the dataset automatically. The cases where the optimization scheme succeeds are therefore more likely to be more morphologically representative, and therefore pre-partitioning the dataset is often counter-productive. Relying on the SVD to extract coherent basis vectors spanning the space of snapshot solutions is therefore a more consistent strategy than performing data triage based on pathological information, under the assumption that the inter-subject mapping can be computed. We emphasize that it is more important to sample solutions from a larger number of patients, than to sample only patients from similar groups.

When applied to the two-compartment tracer model, we obtain relative errors of less than 2% on the REF target patient and close to 3% on the iNPH case when using the largest reduced basis. Meanwhile, on the 7-compartment model, errors are typically in the 9% and 5% range for the REF target patient and 12% to 7% for the iNPH target patient. In many biomedical applications, this error is smaller than the uncertainty due to measurement error. These results are also comparable to works on parameter estimation in the brain, such as Vinje et al. (2023) or Valnes et al. (2020). We also note that the MRI data itself has a relatively low signal-to-noise ratio (Valnes et al., 2020), meaning that the error due to model order reduction would likely not be the largest factor when comparing with measurement data. For many applications involving parameter estimation from data, the results we obtain in this work will therefore likely be within a reasonable range.

### 5.3 Computational costs and speedup

The results in Fig. 11 illustrate the ability of a reduced order approach to significantly accelerate computational experiments. We achieve a speedup of close to 3 orders of magnitude compared to the full order problem. However, the problems we consider are still simple in the context of realistic glymphatic modeling. In applications involving more detailed meshes and more complicated modeling, parallel implementations and specifically designed preconditioners may meaningfully accelerate the full problem. However, the main reason for the speedup in Fig. 11 is likely due to the reduction of the system making it possible to use a direct method when solving the reduced linear system, rather than an iterative one. As long as the reduced basis is small enough to make direct methods feasible, one should expect considerable speedup. We also note that the increase in assembly time to the projection into the reduced space is justified by the acceleration of linear system solver. Even at the largest basis size, the assembly time in Fig. 11(a) increases by approximately 15 s, while the time it takes to solve the linear system goes from 126 s to less than a second, for the REF patient. We also add that when performing multiple realizations of the problem with different parameters, this assembly need only be done once as long as the model depends affinely on the solution operators. However, this online speedup comes at the cost of sometimes considerable offline costs. In our approach the most expensive step is the registration of each patient in the dataset to a target patient. We estimate that the registration takes on average 30 minutes per patient, leading to a total cost of approximately 50 hours for the entire dataset. Solving the full problem, meanwhile, takes at least 3 minutes for the two-compartment model and 100 s for the MPET problem per patient in our implementation. This difference in full order solve time is mostly a consequence of the two-compartment model having a greater number of degrees of freedom than the MPET models, in our implementation. Accordingly, when performing numerical experiments requiring thousands of forward solves, the reduced approach is justified, and significantly more efficient than the full problem. This will often be the case when performing parameter estimation or sensitivity analysis. We may also note that the computational cost of image registration may also be offset by the possibility of using the computed deformation fields for multiple other purposes, beyond our method, in order to compare distinct individuals in the dataset. An additional benefit of the reduced model is the effective compression of any given solution. The full problem may contain millions of degrees of freedom, while the reduced representation is likely to be several orders of magnitude smaller, leading to considerably lower memory demands. The final offline cost to consider when using the POD is the cost of computing the SVD. In general, the computational cost of the SVD scales like $O\left(NM^{2}\right)$ for a problem with N degrees of freedom and M samples, assuming M<N. As previously discussed, sampling more snapshots will mostly lead to better reduced representations, however this may also make computing the SVD considerably more complex. In such cases, randomized methods may be necessary (Halko et al., 2011).

### 5.4 Limitations

As in any data-driven application analyzing medical data from humans, data availability is a fundamental limitation. While brain MRI from 101 individuals is a relatively large number in a medical setting, typical problems involving computational design often leverage the capability of sampling several orders of magnitude more data points for the construction of a reduced representation. In the context of geometry-based brain modeling we will never have access to this amount of data, and it is therefore critical that the brains that can be accessed are also sufficient to allow the construction of an efficient reduced basis. In an abstract sense, any brain geometry is inherently an extremely high-dimensional object with an almost infinite potential for individual variations. However, empirically, we know that brains display sufficiently predictable patterns to enable automated segmentation based purely on $T_{1}$-weighted MRI. In practice, the space of anatomically possible brains may therefore be significantly more compact than one may intuitively imagine. We notice that in the case where the target brain is from REF group in the dataset, the error of the reduced order model, as seen in Fig. 5, Fig. 9 reaches a plateau after a very low number of bases. There is also only a negligible difference between the error obtained from the full dataset with N∼100 subjects and the non-iNPH subset of the dataset containing ∼70 individuals. This indicates that adding MRI of more individuals to the dataset will likely have a minimal impact on the results, to the extent that we are not able to increase the number of available subjects by orders of magnitude.

The other limitation of the present approach is that the brain-to-brain mappings are neither one-to-one, nor unique. We will inevitably not be able to map all points in a mesh built for one brain, to the exactly equivalent point on a mesh built for another brain. Solutions must therefore be extended from the deformed mesh, to the target mesh of interest. A key consequence of the mappings not being one-to-one is that solutions mapped to the target mesh will not necessarily span a conforming subspace of the relevant function space. In particular, we do not expect the boundary conditions to remain respected on the target geometry, as they were in the solution on the original geometry. As a consequence, the space spanned by the solutions mapped to the target geometry ${\overset{ˆ}{V}}_{M}=\text{span}\left({\overset{ˆ}{u}}_{1}^{h}\ldots {\overset{ˆ}{u}}_{M}^{h}\right)$ will not be a subspace of the appropriate Hilbert space $V_{target}$ for the variational problem on the target geometry. This is the main reason why the singular value decays in Fig. 4, Fig. 8 are relatively slow compared to what one might expect for the type of problems we solve in this work. The examples in Fig. 10 illustrate the concrete effect of the inability of the method to capture all cortical folds. In the arterial compartment, the pressure gradient close to the boundary can be relatively large leading to high dependence in cortical shapes. In this case, the reduced method is not able to capture all details in the high-fidelity problem. A remedy to this issue may be to consider nonlinear dimensional reduction methods, such as a variational auto-encoder, which could potentially enforce the boundary condition more consistently than the SVD. However, this approach may prove challenging due to the relatively low amount of available geometries in the dataset.

While this work considers only mappings based on $T_{1}$-weighted MRI, the framework we present is not limited to this imaging modality. In applications where modeling concentration in CSF in the ventricles or SAS it may additionally be relevant to consider $T_{2}$-weighted images. Diffusion-weighted images (DWI) or diffusion-tensor imaging (DTI) may also be of interest for problems including diffusion coefficients which are not constant in space.

Finally, in this work we only consider PDEs which are independent of time for illustrative purposes. However, the present approach extends readily to time-dependent problems. The brain-to-brain mapping is a purely geometrical one, and remains valid as long as the brain geometries are independent of time.

## 6 Conclusion

In this work, we propose a new method for mapping solutions on 3D models of brain geometries between meshes, using medical image registration. We implement an offline-online POD-based approach for building a reduced basis, and apply the method to a dataset of 101 MRI of human individuals. The method is evaluated on two models of human glymphatic function: One problem modeling tracer transport in the brain (Riseth et al., 2023), and a simplified 7-compartment poro-elastic model (Dreyer et al., 2024) of the brain. For each of the two models, we consider one brain from a patient without any identified CSF disorder or neurological disease, considered close to healthy, and one brain from a patient diagnosed with iNPH. In each case, we perform high-fidelity simulations with degrees of freedom on the order of $10^{6}$, and evaluate the error of reduced models of up to 100 basis vectors. We are able to obtain reduced solutions with similar accuracy as state-of-the-art brain modeling methods, while reducing the computational cost of each forward solve to a fraction of the full problem. This work opens up a new avenue for leveraging datasets of brain geometries to build reduced models, an important first step towards patient-specific parameter estimation in glymphatic modeling.

## CRediT authorship contribution statement

**Andreas Solheim:** Writing – review & editing, Writing – original draft, Visualization, Validation, Software, Methodology, Investigation, Conceptualization. **Geir Ringstad:** Writing – review & editing, Data curation. **Per Kristian Eide:** Writing – review & editing, Data curation. **Kent-Andre Mardal:** Writing – review & editing, Supervision, Resources, Methodology, Funding acquisition, Conceptualization.

## Ethics and approvals

Parts of the data presented in this has been presented in previous works on MRI-based assessment of human glymphatic function conducted at the University Hospital of Oslo (Ringstad et al., 2017, Ringstad et al., 2018) in the years 2015–2016. Collection of data analyzed for this study was approved by the Regional Committee for Medical and Health Research Ethics (REK) of Health Region South-East, Norway (2015/96), the Institutional Review Board of Oslo University Hospital (2015/1868) and the National Medicines Agency (15/04932-7), and conducted following the ethical standards of the Declaration of Helsinki of 1975 (revised in 1983). Study participants were included after written and oral informed consent. No new data was collected for the present work.

## Funding

K.A.M. and A.S. acknowledge funding by the 10.13039/501100000781European Research Council under grant 101141807 (aCleanBrain) and Sigma2 — the National Infrastructure for High-Performance Computing and Data Storage in Norway, via grant NN9279K. This work was supported by the foundation Stiftelsen Kristian Gerhard Jebsen through its program for translational medical research.

## Declaration of competing interest

The authors declare that they have no known competing financial interests or personal relationships that could have appeared to influence the work reported in this paper.

## Data Availability

Code for this paper, with scripts for registration and finite element simulations, is openly available from https://github.com/Erasdna/GROM. The dataset used in this paper is not publicly available due to patient data privacy concerns.
